# An Essay on Intermittent and Remittent Diseases, &c.

**Published:** 1829-01-01

**Authors:** 


### AN ESSAY ON THE REMITTENT AND INTERMITTENT DISEASES, INCLUDING, GENERICALLY, MARSH FEVER AND NEURALGIA, &c. By *John Macculloch*, M.D. Two Vols. 8vo.

### ART. III.—NEURALGIA, TIC DOULOUREUX.

WE have now arrived at the second, or rather the third, volume, of Dr. Macculloch's work, (for the volume on Malaria may be considered as the first) and the important subject of NEURALGIA presents itself, occupying the whole of the volume. This distressing malady, or class of maladies, divides itself into so many forms, and assumes so many questionable shapes, that it will require two or more extensive articles to trace it through its ramifications. Nor do we think the pen of author or reviewer can be more profitably employed than on such a task.

It is now a considerable number of years since Dr. M. treated neuralgia as a mode or variety of intermittent—a doctrine not generally, if at all, adopted by the profession at large.

"In the disorders which I am about to enumerate, some have been considered as independent diseases, arising from very different causes, while, of these, there are a few which have even established an independent character almost as antient as physic itself.

"Others have been viewed as symptoms of some disorder, but not of this one; while others again have been considered as trivial, or else inexplicable; mysterious cases occurring once in an age, and out of the common rules of physic: while, still further, a few have been viewed as the produce of external injury; mechanical solutions of continuity or derangements of structure. Under such a confusion of names and opinions, I had no resource but to form an entire new arrangement, by creating apparently-new diseases and forming new terms, or else to abolish old and received diseases with their names, treating them as varieties, or lastly, at least presuming to treat as Neuralgiæ, under their old names, those varieties which were already named, and to suffer the others to remain as nameless as they have hitherto been. And this plan, as the least offensive, I have adopted." 5.

### I. NEURALGIA OF THE FACE—TIC DOULOUREUX.

Heberden and Fothergill appear to have been the first who allude to the disease in this country, though the term tic douloureux originated with André, a French surgeon, who also proposed the division of the nerve, in 1756. The public then considered it a new disease—and still think it a rare one:—"But it is neither new nor rare." The term alone is new. The apparent rarity is thus accounted for by our author.

"To be noticed as a case of this disease, the pain must be excessive, and must also be limited to a peculiar part of the face; and further, it must be found in the opulent, or in those who, little accustomed or willing to bear pain, fly to physic for relief, and thus call attention to themselves and their cases. There are thousands who suffer from it, under forms less marked; and thousands, in the middling and lower classes, who endure it, even in its worst form, but of whom the public never hears. He who will thus seek it out, will soon be convinced of the truth of this assertion, as I have long since been : and though he may find a much greater number of the cases not decidedly marked by the strongest and most peculiar features of this disorder, and therefore not known by this new popular term, he will be singularly unfortunate if he does not also meet many, of the best defined and most intense character, generally endured as best they may, and seldom forming objects of attention to the great mass of practitioners." 8.

We are quite convinced of the truth of the above representation. In a single, and not very extensive tract, in this country, Dr. M. has been able to collect not less than a hundred cases among the labouring classes. The names under which the disease formerly went, before the term neuralgia came forth, were probably rheumatism of the face—affection of the antrum—clavus hystericus—tooth-ache—periodical head-ache—wandering gout, &c. The term neuralgia is certainly more scientific than tic douloureux, and more useful, inasmuch as it points out a road to a generalization, which has certainly been slowly taken advantage of by physicians. In this place, Dr. M. passes a just censure on that system of sparring which has lately been kept up between the medical and surgical branches of the profession, as if there was any real division in nature between the two. But the physician is taught to abhor the knife—while the surgeon despises the Pharmacopœia. Dr. M. accuses surgery of keeping back a knowledge of neuralgia, by the attempt to insulate the disease from its proper connexion with the general health—in other words, to cure it by the scalpel. The division of the nerve, however, is now so universally given up, that we need not waste time on its condemnation. That the pain of neuralgia is not confined to any particular nerve, or class of nerves, admits now of no doubt—not even excepting the ganglionic nerves.

"I need scarcely say, that, in this disorder as it stands in the popular acceptation, the pain is most severe ; being probably as great, often, as the nervous system can endure : since all the surrounding objects sometimes disappear before its violence, or it deprives the patient of all other sensations ; in vulgar language, of all his senses ; frequently also inducing absolute delirium, as I just noticed ; an effect rarely occurring from any other pure pain than the inflammation in the bowels. This extremity of pain is commonly instantaneous in its attack ; resembling very much, in this respect, the electrical shock ; and most generally it is similarly transitory, coming on in repeated fits during the continuance of the entire paroxysm. It is not, at the same time, always the sole pain ; since a general painful state exists through the whole paroxysm ; sufficiently severe, should it be the only one, as it often is, but appearing almost pleasure, when contrasted with the more acute shocks of the nerve." 16.

During this state, the integuments, or rather the whole surrounding parts, become extremely irritable, or tender to the touch—generally more so from light than from firm pressure. It is not unusual to find a vascular turgescence accompanying the pain, which might, *à priori*, be expected, on the principle, "ubi stimulus ibi fluxus." This turgescence, indeed, sometimes amounts to a species of inflammation, resembling that of rheumatism—an analogy that it may be useful to bear in mind. As the pain ceases, the general soreness rather increases, and often remains for a good while, leaving the patient in comparative health, or, more frequently, in a state of debility.

"Such is a simple and ordinary case of a paroxysm of this Neuralgia ; but many accessory circumstances of an equally obvious nature sometimes attend it. Thus, the neighbouring muscles, and even the more distant ones of the limbs, are often affected with spasms, occasionally to a considerable extent; while, not unfrequently, with the usual charity of the healthy, they are attributed to impatience and debility of mind on the part of the patient; as is more especially and certainly the case when these spasms seize on the larynx, producing involuntary cries, or converting the ordinary breathing into audible sounds. In truth, no one is entitled to assume the office of critic on a Neuralgic patient, who has not been a patient himself; and, for the cause of charity, it were perhaps to be wished that the disorder were more common, or that a short apprenticeship at least to it, were a more general lot.

"Frequently also, there is an increased flow of saliva, and, much more often, of tears, which, in some cases, almost stream from the eyes; scarcely noticed by the patient, but not so by the by-standers, always ready to attribute to mental weakness, what, in this case, is a mere increase of secretion from disordered excitement of the glandular nerves; forming, in fact, a peculiar variety or modification of Neuralgia which I shall have occasion hereafter to point out more distinctly. And if it is an episodical remark, I know not where better I can introduce it than here; that in patients who have suffered severely from Neuralgia under any of its forms, if the lachrymal glands have once been materially affected by the disease, they rarely recover their healthy state: and thus, that tears continue to be, not only easily excited in those who scarcely knew, before that, what it was to shed one, but that they frequently occur, and even in streams, without any mental cause at all, and not uncommonly in sleep, though no dreams are present, or at the hour of awaking; often lasting a considerable time, and producing no small inconvenience." 18.

Lastly, there is often a sympathetic affection of other and distant nerves, where anatomy can trace little connexion, except through the medium of that common centre, the brain. The ingenious author now goes beyond the surface of things, and examines into certain less obvious characters of the complaint, which help to shed light on the real nature of the malady.

"Immediately before the attack, if the pulse is examined, it will be found to put on that character which it possesses in the cold stage of an intermittent; while, through the progress of the paroxysm, it passes through the other analogous changes. If also a watchful patient, at least when directed to do so by his physician, (which I fear has rarely been the case,) attends to his previous feelings, he will find that there are most commonly some indications of a cold stage, generally obscure, it is true, as is the case in most of the anomalous and chronic intermittents, but still discernible: while doubtless it may sometimes be wanting or difficult to make out, as is so often the case in such obscure intermittents. When most distinct, it is like the sensation of cold water applied to some part of the face, or trickling over it, being indeed often thus described by patients; or there may sometimes be a sensation of cold, more general, if also transitory. The skin, at least of the face, also becomes pale and shrunk, with that peculiar physiognomy attending ague, so indicative of all these diseases, if so perpetually overlooked: and this is a symptom which, if unnoticed by the patient, ought never to escape the eye of an observing physician, explanatory, and often useful as it is. Occasionally, this paleness is local instead of general; and I have seen cases where I could pronounce that the paroxysm was threatening, from one side of the face turning suddenly white while the other retained its natural aspect and colour.

"If this is the cold stage of this particular intermittent disease, the fit of pain appears to belong to the hot one, or thus at least has it always seemed in my experience: and if, as a hot stage, it is not a very marked febrile state, it is sometimes sufficiently apparent, in an increase of heat, local if not general, in the change of the pulse, and in a thirst which occasionally accompanies it. And if it is a slightly-marked hot fit, it is not slighter than that which often occurs in chronic intermittent when other kinds of local symptoms are present, or even when the cases are pure; while in such cases, as well as in this and other Neuralgiæ, the sweating stage is rarely well marked, or is perhaps only discovered by the facility with which that effect is produced by exertion." 21.

Thus Dr. M. has described a paroxysm of neuralgia as resembling that of an obscure or chronic intermittent, with the superaddition of its peculiar pain, or local affection of a nerve. It yet remains to examine what resemblance the general course of the disease bears to the common or simple intermittent.

There are two forms of this, as of other neuralgiæ—the acute and chronic—new or habitual. The chronic and habitual is, like all intermittents, far less regular than the former. In the recent state, the pain returns in distinct and defined paroxysms, leaving an interval of health. These returns are generally quotidian, rarely tertian or quartan. But when the disease is of long standing, the regularity is much less distinguishable than even in common or anomalous ague—" with the single exception of the affection of the heart." This irregularity, however, may be more apparent than real. How seldom are complaints accurately watched, and faithfully recorded. Sauvages relates a case where the pain recurred every eighth day, and lasted 30 hours. It was in the temporal muscle, and was denominated by Salius the *hemicrania lunatica ;* the entire duration of the disease being three years and a half. Why the recurrences of neuralgic affections should be less regular than other kinds of periodical disorders, our author is unable to explain. The PERIODICITY not being expected, is probably not observed or noticed as carefully as it should be.

" Even should the nerve be affected at the habitual period, that affection is not always a fit of the violent pain. On the contrary, it may be a very slender one, scarcely noticed by a patient used to much greater suffering ; or it may appear as a toothach, or a headach, or what is called a rheumatic pain of the face : and thus, although really a paroxysm of the disease, may deceive both patient and physician ; and most of all when, as in the cases last enumerated, it will admit of some common or vulgar name. Such in fact is the character of the Neuralgia, in whatever place situated : and the fundamental error here has been founded on that to which I formerly alluded, namely, the assigning it a name expressive of violent pain, and the separating this particular variety from all its analogies and variations. Such are the endless errors produced by ill-chosen terms, and by giving to such terms an improper value. And still further, as I shall hereafter more decidedly show, as this disease is but a mode of the intermittent, it may be exchanged for or replaced by a common paroxysm without any local pain ; while even that paroxysm may be so slight as to attract little notice from any one, and least of all from a patient accustomed to much keener sufferings." 26.

When of long standing, however, it becomes, like every other chronic intermittent, a truly irregular disease. It may come as a single attack in the midst of weeks or days of repose, excited by some occasional cause—or it may, in the same way, adopt any modes of irregularity.

Such is the general character of the facial neuralgia—a character that applies to all the varieties of this, as well as of the other neuralgiæ.

### VARIETIES.—I. PERIODICAL HEAD-ACHE—VERTIGO.

This disorder is of such frequent occurrence, that every man's experience must furnish abundance of examples. Its periodicity is acknowledged—it is generally cured by the remedies employed for ague—and yet its connexion, in an etiological point of view, with the latter disease, is little, if at all suspected.

" I could quote," (says Dr. M.) " physicians of the highest note, who had submitted to it in their own persons for a long life, even in its most marked and regular form, the tertian, without adopting the obvious plan of cure, and even arguing to the last against the present view of its nature." We think they must have been desperately fond of a disagreeable companion, who would neglect bark, arsenic, and change of air, on such an occasion. Dr. M. considers the present disease as a connecting link between ague and tic douloureux—more nearly allied to the former than the latter, as being void of that acute and dreadful pain, so characteristic of the high grades of neuralgia. The periodical head-ach, Dr. M. observes, may possess any of the types of intermittent. He has seen it as a double quotidian, as a single one, and as a tertian. He has never said that all the disorders collected under the head of intermittent, must, of necessity, arise from malaria—though he has tried to prove that this poison is often present where it is not suspected. We are certain, only, that malaria is by far the most frequent cause of them. Indeed, it has never been proved that a simple ague will *originate* without this cause, though it may be readily excited afterwards by many causes.

"But when we descend from the simple disease along the scale of varieties, or as the local affections of the nerves begin to predominate far above the general one, I am so far from thinking Malaria essential to the production of intermittent, or, to speak specially of Neuralgia, that I have made a particular division, where it is demonstrably produced, as far at least as we can demonstrate, by an exclusive cause, even by mechanical injuries." 32.

Dr. M. therefore, does not say or insist, that periodical head-ache is necessarily or exclusively the result of malaria—though this may be frequently suspected to be the case. Thus, in Spain, the *migrania* is endemic in all marshy situations throughout that country. But, even were it proved that the disease in question was produced by other causes than malaria, this would not affect the theory of its common or generic nature with intermittent.

"The action of Malaria is on the nervous system, on the whole and on the parts: or, affecting the whole in a slender manner, it may exert its chief influence on some peculiar nerve or portion of a nerve. And till we know more of their nature and of this action, we cannot pronounce a negative, or say that no cause but Malaria shall produce even a simple intermittent: while, if mechanical injury of a nerve can produce a partial one, there may be many other causes, unknown to us, capable of generating a periodical headach." 33.

Be this as it may, the malady in question, is most common in situations of the unhealthy kind already often described—and is frequently brought on directly by the causes which produce intermittents. A still stronger fact is, that it most commonly occurs in those who labour under chronic intermittents or remittents—or who have formerly suffered from those disorders. Still more remarkably, it is found to interchange by paroxysms, with the common intermittent—or the ordinary fever of one day is replaced, on another, by the headache.

"Thus have I seen the headach and the ague-fit occupying the alternate days, a modification which may be considered as a double tertian, and proceeding thus through a long period; while the same species of combination has also occurred to me under the tertian type. Where the chronic intermittent, however, has been of long duration, it is more common for the periodical headach which is united with it to recur in a very irregular manner, as is true generally of the disorder in all its modes, when of such standing: and it is in these cases especially, that its real nature is so commonly mistaken, as it then wants those obvious characters which alone would arrest the attention of a superficial observer." 34.

Dr. M. thinks that the pain in the neuralgia, and all anonymous intermittents, appertains to the hot fit—" and if an absolute general hot stage does not occur, there is that excitement of the vessels, and local heat, often approaching, or even amounting to temporary inflammation, which marks, at least a hot fit—a hot stage, as local as the previous cold one commonly is, just as if the whole intermittent paroxysm was limited to one spot, instead of occupying the whole nervous system." The characters are, in short, those of an ordinary intermittent, with the addition of pain—proving the correctness with which it is arranged as a neuralgic intermittent.

"In the best defined periodical headach, the pained part is generally small, when it may exist in any spot, including the face; and it is a general rule, though not without its exceptions, that in the chronic cases, whatever spot is once the disordered one, it continues so throughout the whole career of the disease; indicating a permanent morbid condition of some nerve, as in the decided Neuralgia of the face. And thus, as these cases become severe in point of pain, do they approach so gradually to that Neuralgia, that no boundary whatever can be drawn between them; a fact which ought long since to have explained what the Neuralgia (Tic) really was." 40.

These localities (of periodical head-ache) are sometimes so small, that patients say they could cover it with the point of the finger—another character in which it agrees with the TIC. Thus, it is not uncommon, over one eye-brow—or on the top of the head, and so on. Dr. M. has met with no case of clavus hystericus which was not one of the disease in question, though he denies not, that the former may be, sometimes, an original and independent affection.

Hemicrania, another modification of the disease now under investigation, sometimes occurs under a form so slight, that it attracts no notice—at least from the physician—a circumstance, however, that tends to explain some of the errors respecting this disease. Thus, a paroxysm of it, even of a regular and persistent form, may occur as a mere sense of fulness or heat in one side of the face, often attended with lachrymation, or a slight redness of the corresponding eye, or temporary redness of that nostril. This kind of attack is mistaken for cold, weakness of the eye, &c. and its true nature overlooked.

"I must yet, however, notice one point of resemblance between the common intermittent headach, and the Neuralgia (Tic) of the face. In the former, as in this, there are often two pains, an acute and a general one, at the same time; while the acute pain also is subject to similar intermissions and exacerbations during the paroxysm. And when the disease is strongly marked, or the pain intense, the confusion of thought formerly noticed frequently amounts to absolute delirium, as the whole disease is commonly attended with those affections of the mind or temper, which were formerly enumerated under simple intermittent fever. Thus further does this disorder, as well as the Neuralgia under whatever form, frequently excite the desire for suicide, while the very act itself has been committed; of which I shall hereafter be compelled to notice one peculiar case." 47.

The effects of the neuralgiæ on the mental faculties are analogous to those of intermittents. Hebetude, tendency to fatuity, and other phenomena of mental debility, are among the consequences of long-continued periodical head-aches, and indeed most of the severe neuralgiæ.

Dr. M. takes an opportunity here of ridiculing the fashionable term of "determination of blood to the head."

"In the case to which I allude, it has been recently discovered, (for the disorder is of very recent invention) that the blood flows in some very improper manner to the head, even should the patient be a delicate and young female, a pallid and enfeebled, night-watching student, a nervous lady of fashion, exhausted by London vigils, or any one else of all those who were once esteemed to suffer from debility and nervous diseases; a tribe too numerous to mention in detail. Who was, or who were, the enlightened discoverers of this new philosophy, may be asked by those who can themselves answer it: a physician must hope, for the honour of his profession, that it was the discovery of the cuppers, and that it has been propagated by the self-empirics who are now fast becoming the rivals of his brethren, in the science, and of the apothecaries, in the art." 53.

These symptoms, formerly called nervous, and still looked upon by many as sympathetic, are very frequently, according to our author's experience, "the produce of the intermittent affections of the head," which have been described —or are actually cases (if sometimes obscure ones) of the periodical head-ache —"disorders of a neuralgic character." We fear that the real nature of these "determinations" to the head, is very often mistaken, and that lancets, leeches, and scarificators, are not seldom put in requisition when they might safely be dispensed with.

### II. NEURALGIÆ OF OTHER NERVES, IN VARIOUS PARTS OF THE BODY.

The minuteness with which our author has described the neuralgiæ of the facial nerves will render it unnecessary to be very minute with other descriptions. Dr. M. seems to have made a mistake in supposing that any doubt remains in the minds of medical men generally as to the extension of neuralgia to other nerves of the body besides the face. This being the case, we need not waste time in dwelling on the proofs of that which we, at once, admit. The proofs of identity, or at least of strong affinity, between the neuralgiæ generally and intermittent fevers, are, of course, drawn from the same sources as those had recourse to, when endeavouring to assimilate tic douloureux with ague.

"Very often we can trace it to the same causes; and while it is cured by the same general remedies as common intermittent, with the addition of such local ones as are found of use in the Neuralgia of the face, it is exasperated or rendered permanent by that which is equally maltreatment in all those diseases; while also the very pointed evil consequences produced by the evacuant and debilitating practice, are the very same or exactly analogous to what they are in Intermittent and in acknowledged Neuralgia, whatever be the place of the pain. Further, these pains, or painful disorders, be the situations what they may, alternate with common Intermittent, in the several ways already described as occurring in the local anomalies of that disorder, and in Neuralgia and periodical headach; that is, as whole periods, or as mere paroxysms: while, of course, they are also irregularly intermixed with such affections, as happens with respect to the whole of the disorders treated of in this essay, whenever they are of long duration or have become an inveterate habit.

"Thus also, in a patient of this nature, it is not uncommon to find a very great number of all the disorders that have been here enumerated, co-existing or succeeding, and in every possible mode of combination and succession. The same patient, for example, who has suffered common intermittent, will be found to have also experienced more or less, or even the whole, of the anomalies enumerated under that disease, together with the Neuralgia of the face, the periodical headach, and a certain number of the neuralgic diseases which I am about to record; all these several affections alternating in different ways among each other, and many being occasionally united; while, what renders the proof of the nature of these last complete, is, that whatever period is the habitual one of the commonest intermittent paroxysm, the same will mark the recurrence of every one of the affections in question. Such a patient, (and I have seen more than one such,) becomes in himself a perfect nosology of this disease, and carries in his own person a demonstration that ought to convince the most incredulous. Lastly, I have never seen a case of these remote and less common Neuralgiæ, where it was not easy to trace that febrile state, which, however slender it may often be in all the local affections, is never absolutely wanting, and ought never to escape the eye of a real physician. The pulse undergoes the same changes, the fit of pain is the hot stage, and the cold stage may be found, at least by means of those delicate tests which I have already pointed out: nor have I ever been introduced to a patient reported as labouring under some unknown and extraordinary painful disorder, that I could not pronounce at once, from the mere physiognomy, (taking care to be present before or at the accession,) what the disease was ; provided of course that it was really a neuralgia, as, in truth, has almost invariably proved the fact." 76.

Dr. M. proceeds to the statement of some insulated cases, which, though apparently anomalous in their character, yet bear on the subject under investigation.

Optic Nerve. A patient described the pain in this part, as though a red-hot needle had been passed through the centre of the eye. " As this disorder accompanied an attack of neuralgia in the upper jaw, or rather replaced it, appearing at the moment when that ceased, and as it was cured by arsenic, there was no reason to doubt of its nature."

Testicle. " Of this I have known, personally, but two cases ; while I have reason to suspect that it has occurred frequently, and been mistaken for an incipient scirrhus. In one of the cases to which I allude, this error was in fact committed ; and after a long period of suffering, the gland was extirpated in the usual manner. It was found to be sound, and, as generally happens when the division of the nerve has been resorted to in the neuralgia, it returned in the cord. This case was known to me, only after this last event ; and as the patient was an opulent one, there had been no want of advice respecting the disease. It must be hoped that such mistakes will now become less frequent ; while I must not even allude to the place where this one was committed.

" The other case, under my own care, was immediately cured by arsenic ; while its nature was rendered perfectly evident by the slight paroxysm of intermittent which attended it, and by its having alternated with another Neuralgia. As might be supposed, the pain in this case was extremely violent ; and it was described by the patient as rendering him entirely blind, (as he expressed it,) to the surrounding objects ; as if the whole world had disappeared from his sight, and all recollection was obliterated. Had such a pain continued even for a few minutes, it must have produced delirium." 78.

Hand and Fingers. Dr. M. has seen many cases of this nature, but describes only two, on account of the particular circumstances attending them. In one, a patient who had suffered from chronic intermittent and neuralgia in various forms, the affection was in a small branch of the radial nerve which runs along the metacarpal bone of the fore-finger. The usual symptoms were present ; but the peculiarly superficial position of this nerve enabled Dr. M. to detect a change of structure in it which can seldom be observed, " and which is probably always present, though it has been little suspected." It is a pity, we think, that the precise nature of this change was not determined by the excision of the nerve—an operation proposed by the surgical attendant. The pain was limited to a space which a pea could have covered. In the course of four months, during which the neuralgia lasted, the nerve enlarged so as to form a knot or swelling of similar dimensions—" or, more accurately speaking, about a sixth of an inch in diameter." This part was so extremely sensible as not to bear touching even with a feather, without exquisite pain. This swelling continued after the neuralgia disappeared, but gradually lessened in size, though it has never (after a lapse of eight years) entirely subsided or lost its morbid sensibility. The paroxysms of pain were, in this case, periodical, and attended with redness of the surrounding parts. Blistering invariably increased the malady, and enlarged the circle of the pain and irritation. This is an effect which our author has generally found to follow blisters in neuralgia. Several other curious and obscure cases of neuralgic affections of the fingers are related by Dr. M. but we must pass them over.

KNEE. In these cases of neuralgia of this joint, the disease was pronounced to be scrofulous, and to threaten white-swelling.

" In one of the cases, it had lasted five years; and as the pain was very severe, the surprise had long been that no swelling could be discovered by the touch. Had these remote Neuralgiæ been then suspected to exist, or had the disorder at large been understood, as I trust it will now be, it could not have remained a subject of doubt for as many days, since the attack was quotidian and accurate; and it is one, yet but one out of thousands of cases, which shows how necessary it is that the knowledge of this disease should be universally spread among practitioners, since the quantity of human suffering that has already resulted from that ignorance is incalculable. I need scarcely say that, in this case, gout and rheumatism had been resorted to for the solution, when, after some duration, no swelling or disease of the bones could be discovered.

" But one further remark on it is worth making. The pain had always been severe; and from that, and its long duration, the constitution of the patient, a female in the better ranks of life, was reported by the attendant practitioner, to be 'broken down' by the suffering. A mere glance however was sufficient to shew, not only that chronic intermittent was present, but that she was labouring under visceral disease, probably in the spleen; and thus it frequently happens in these cases, wherever they occur, that such a state is ignorantly attributed to a 'broken down constitution.' It is scarcely worth while perhaps to add, that the pain soon yielded to bark and arsenic given alternately; but many more months, added to change of air from the unhealthy and marshy situation where this patient resided, were required to quell the simpler intermittent which attended it.

" Of two other cases in this part, it is worth while to remark that the extremely acute pain was situated over the very edge of the head of the tibia, yet between the harder parts and the skin, as the patient determined it; while the affected place was not more than the eighth of an inch in dimensions. As scarcely any accuracy of dissection could produce a visible nerve in this precise spot, it follows that a perfect and intense neuralgic pain may exist in the most minute branches; a fact which has occurred, in my experience, in other parts; so that the want of a demonstrable nerve must not be used as an argument against the existence of the disease generally, any more than it must be permitted to blind the practitioner as to its possible presence in any individual case of difficulty.

" The last of these cases which I think it worth while to notice, was remarkable for its double tertian, or alternating, form; a fact not very unfrequent under different modes, and which I have noticed elsewhere. I think, at least, that the term double tertian may be applied to it, from its resemblance to that mode of the simple intermittent in which the two fits are dissimilar, and from its analogy to those cases, not uncommon, in which a fit of Neuralgia and a paroxysm of common intermittent occur on alternate days. And it belongs to that class of cases in this disorder, which aids in establishing the common generic nature of all these diseases; being one of the links which connects the most simple and regular intermittent at one extremity, with the most obscure Neuralgia at the other.

"In this case there was, on one day, a pain in both the knees, and, on the alternating one, a pain in one arm; and thus had the disorder lasted a long time, to the great discomfiture of the physician by whom I was introduced to it, and who, as might perhaps be conjectured, had considered it as an irregular gout." 87.

Our readers will not fail to perceive that the above is what surgeons have lately called "HYSTERICAL WHITE SWELLING"—more especially Mr. Brodie, and the surgeons of St. George's Hospital. In a late clinical lecture on this subject, Mr. Brodie stated it as his opinion, "that nine out of ten of those unfortunate young women who have been *doctored* (á la Harrison of course) of late years, for '*spinal diseases*,' have really laboured under nothing but *hysterical pains* in the back." In the same journal (the Medical Gazette) there is a curious case detailed of "nervous affection" of the breast, of which the following sketch may be appropriately introduced here.

"The left breast, to the view, was no larger than the right; indeed, on the whole, rather smaller; the skin had the least blush of redness upon it. On examining the gland it was felt to be traversed by a distinct and circumscribed hardness, dividing the upper portion of the gland from the lower. The indurated part was exquisitely painful upon pressure, especially from the nipple outwards to the arm-pit. Pinching, or even lightly tapping on the skin, in the same situation and direction, gave pain, nearly as much, indeed, as firm compression. As the examination receded from the before-mentioned line, the pain and the tenderness, *pari passu*, diminished. In the right breast were several indurated spots; much pain upon pressure; and great sensibility of skin. The pain was not aggravated but relieved at nights; sleep difficult indeed to obtain, but when obtained sound and unbroken. Besides the affection of the breast she had pain in the side, brought on and increased by exercise or motion. Her appearance was healthy; the appetite good; the tongue moist, but white; the bowels confined; the menses irregular.

"For the right understanding of the case it is necessary that we should mention the leading features of hysterical pain, at least in the external parts of the body. First, it is as much increased by slightly tapping, or at least pinching the integument, as by actual and considerable pressure. In the second place, the sensibility is not only exalted but actually exaggerated, caricaturing, as it were, the pain of organic disease. Thirdly, it differs from the pain of inflammation in this, that though it may prevent the patient's going to sleep, it seldom or never wakes her from it. This, according to Mr. Brodie, is a decided characteristic of nervous pain. In the present case these symptoms were observed, combined with induration and chronic inflammation in the gland of the breast."*

In respect to the name of the disease in question, whether seated in the knee, the mamma, or any other part of the body, we decidedly prefer the term NEURALGIA to that of HYSTERIA. The latter means nothing, in these cases—the former not only designates the nature of the complaint, but points to the etiology, and even to the treatment.

TIBIA. Many cases of neuralgia of this part have fallen under our author's notice. They were generally mistaken for periostitis, (and why may they not have been occasionally combined with inflammation?) and were treated by mercury and sarsaparilla, under the idea that they were of syphilitic origin. This treatment often succeeded, because mercury and sarsa are usually beneficial in neuralgia, and these cures would, of course, confirm the practitioner in his erroneous view of the disease.

---

* Medical Gazette, No. 45.

RECTUM. Of this Dr. M. had an opportunity of seeing one well-marked case, and of observing its phenomena very minutely. He had to maintain his opinion against almost an army of medical men—no one, at that time, conceiving that neuralgia could be seated in any other part than the face. The case in question is a very curious one, and we are tempted to give it in the author's own words.

"At the first attack, and for some weeks, it consisted in an occasional sensation like a spasm, apparently situated in the urethra, about the prostate gland, recurring three or four times a day, and causing little uneasiness. Gradually, these sensations increased in frequency, and were attended with a general sense of irritation about the neck of the bladder, very much increased by walking, and at length producing spasms in various parts, with a tendency to an hysterical paroxysm. No apparent fever of any kind was at first present; nor any suspicion of its real nature entertained; while the disorder, not yet strictly periodical, was referred to the urethra and bladder. Very shortly, there supervened a debility, with occasional numbness, in one leg; and it was easy to trace, by the tingling sensation formerly described, the course of the fibular nerve. At the same time also, it was perceived that the mere act of bending the neck forwards, brought on the sensation in the perineum, and further, caused the patient to totter on the affected leg; a circumstance to which I shall have occasion to recur hereafter.

"After enduring some weeks in this form, there supervened a febrile state, at first very obscure, but which was, after some time, ascertained to be a double quotidian with a nocturnal and diurnal paroxysm. Still, the nature and the real seat of the pain was obscure; nor was the slightest suspicion of Neuralgia entertained by the numerous medical attendants who in succession examined the case. Such however it appeared to me; and after some further progress, every doubt in my mind, if not in that of others, was removed by the increased regularity of the disease, and by the pain in question becoming as regular as the attacks of Neuralgia are when most perfect. In this state, the first of the quotidian paroxysms was simple, and the second was attended by the Neuralgia, which, now increasing in decision, increased also in severity. In this aggravated state also, it became plain that the primary seat of the pain was in the rectum, the patient describing it as a burning heat, as from a heated solid introduced, which was shortly communicated to the bladder, producing irritation and strangury. When of this severe nature, that irritation extended even round the thighs and over the lumbar region; so that the slightest touch produced great uneasiness, as happens in violent cases of the Neuralgia of the face, and was felt even in the abdomen, as if the whole colon was affected in a similar manner; which was probably the fact.

"Further, during the severity of the attack, all the limbs were affected with spasms, and very generally there supervened a regular fit of hysteria, with a great degree of general derangement throughout the whole system, consisting of the usual symptoms of a severe remittent in all their worst forms. Lastly, and not to be unnecessarily minute, as the irritation of the bladder appeared to spread along the ureters to the kidneys, there came on diabetes, the diabetes mellitus; while, when this symptom was peculiarly severe, it was attended with an acute pain in the left, but not in the right kidney, so that possibly this particular symptom was confined to one of the glands only. And respecting this part of the disease, I must further add, that it was rigidly paroxysmal, or that the morbid secretion of sugar commenced with the general fit, and entirely disappeared in the interval." 95.

The patient had been subject to intermittent, and also to neuralgia, under various forms, for a long course of years —" and this was one of the relapses— a relapse which was often afterwards repeated." Although with the repetitions, the severity of the disease abated, yet, " on no one occasion did the period of the attack, and the continuance of the pain, vary, by even half an hour, from those first established." The cure of these relapses—or the mitigation of their severity, was effected by the remedies which succeed in neuralgia and chronic intermittent—and the disorder was brought back by every debilitating cause.

*Thigh.* Dr. M. has seen many cases where the neuralgia was seated in the thigh—especially in the anterior crural nerve, at the exact flexure of the thigh—and also in its ramifications as low as the middle of the limb—in the former case, shooting, as sciatica does, and with similar severity, down to the toe. Although the paroxysms were not regularly periodical, yet there was always a period of the day, in which the neuralgia was wanting—and this period was always the same.

*Kidney.* Dr. M. has met with but one case of pure neuralgia in the kidney, unattended with diabetes, or the increase of urine.

SCIATICA. This species or form of neuralgia has perhaps attracted more attention than any other—probably on account of the great severity of suffering which it occasions. It gives our author a fresh opportunity of satirising physic and its professors, in respect to the vague manner in which the science, if such it may be called, has been prosecuted. Thus, Cullen and others have ranked sciatica with rheumatism.

"Why was it ranked with Rheumatism? because it was a painful disorder: such are the reasoning processes of physic, yet it complains of difficulties. And what is Rheumatism? a painful disease also; 'a peculiar affection,' says Cullen, 'of the *muscular fibres,* with an inflammatory disposition in the system at large.'" 110.

Dr. Cullen having thus classed it with *muscular* affections, passes over it as unworthy of regard—"proving further that he looked upon it but as a variety." Nevertheless it was long ago discovered that sciatica was seated in the nerve, though this led to no results—and Cullen treats the opinion and its author with neglect.* Living writers and practitioners consider sciatica as seated in the nerve. Its theory has been derived from the neuralgia of the face—and, in short, it is tic doloureux of the sciatic nerve. In looking over ancient and foreign authors, we find notices of sciatica attendant on intermittent fever, or succeeding it, and of the same disease having periodical returns with or without intermittent. There are even cases, (as related by Werlhoff, for example) where bark was administered, on those grounds; but still the disease was never placed in its proper position as a neuralgia, till recently. Even now, our author thinks, little has been gained, in consequence of the error committed by the attempt to connect it with *inflammation* of the nerve, or its covering—an error which "can only lead to wrong practice."

"It is on this view of its nature, that we can explain the peculiar intensity of the pain; a pain, both for quality and violence, bearing no resemblance to that of rheumatism. On this view also, we can explain its confined nature, the absence of inflammation, the state of diffused irritation about the parts which attends the fit of pain, the shocks which accompany it, resembling those of all the Neuralgiæ, and its propagation along the course of the nerve. And if these peculiarities are thus explained, so are they the proofs of its real nature; while similar and further proofs are found in the paralytic affection which so often follows it, and which, as I have already shewn, and shall more fully show hereafter, is a frequent consequence in all

* Cotunnius on Ischias Nervosa.

Neuralgiæ, particularly under bad or wrong treatment. If spasms in the adjoining muscles, or throughout the body in general occur in sciatica, so do they in all the Neuralgiæ; and it is further remarkable that it produces, in irritable habits especially, hysterical symptoms, as do the other disorders of this nature, together with a general irritability, both of mind and body, which seems to belong peculiarly to the painful diseases of this nature." 117.

It will be objected, adds Dr. M. that sciatica is not so strictly periodical as this theory would demand. But what has been stated respecting other neuralgiæ will probably apply to this—the periodicity has often existed without being remarked or recorded. But there is no want of recorded instances of its periodical character. An acute medical friend of the author, "residing where this disorder is endemic, now constantly observes this character, which, till pointed out to him, he had overlooked, or, in fact, had never once perceived." He now seldom fails to discover, also, the intermittent fever, even regular, when he finds it to connect itself, as to the place of its endemic occurrence, with neuralgia of the face.

"When further he writes to me, that since he adopted arsenic at my suggestion he seldom fails to cure it, and lastly, when we know that in the ancient cases, change of place or air is the best remedy, I can scarcely believe but that when these opinions shall become more universally spread, I shall see the same testimonies from many other quarters, and that this most painful disorder will no longer be that opprobrium to physic which it so long has been." 119.

In fact, if the absolute paroxysmal and intermittent character of sciatica is not frequent, it seldom happens that a distinct exacerbation is not to be found—though this is generally overlooked, in consequence of the very vulgar philosophy, of the patient's—"becoming worse when he is warm in bed"—in other words, when the nocturnal paroxysm obtains. In short, our author avers that he has never failed to recognize "the intermittent febrile paroxysm, under all the characters or variations which attend it in neuralgia in general." By this, Dr. M. does not mean to infer that sciatica is always the product of malaria, since he has already shewn that the other neuralgiæ often arise from simple causes—even from local injuries.

"It is observed to be peculiar, in a general way, to certain situations, or to be a sort of endemic, in those, while little known in others; and thus for example, it is extremely common in Cumberland and Westmoreland, among the peasantry. It is a singular analogy to this, that, in a certain district in Wales, the Neuralgia of the face should be the common form of the disease, while the sciatica is, comparatively, little known; but how much further such facts may extend, we have as yet no information; and for the plain reason, that we have had no observers and no philosophy. I do not pretend even to imagine a cause; but, if it is of any value, or rather if it may ultimately be of any value when our knowledge of these diseases shall become more extensive, there is something similar to this in the common intermittent, as I formerly observed; since there are certain situations which produce quotidian or tertian, or quartan, in preference, respectively, to the other forms. And if what I have attempted to prove in another place be true, namely that the enlargement of the Thyroid gland is caused especially by the Malaria of alpine valleys, here is another fact and an analogy, seeming to prove that there are distinct modes of Malaria productive of distinct disorders." 124.

*Neuralgiæ of the Glands.* Passing over a chapter on "Questionable Neuralgiæ," which deserves perusal, but on which we dare not linger, we come to a chapter on neuralgic affections of glands. Considering the abundant supply of nerves to glands, we need not wonder that the functions of these organs should be disturbed by neuralgia. An abundant flow of tears and saliva has been observed in neuralgia of the face. In some cases of tic, or head-ache, there is a copious discharge of water from the nose. Dr. M. has known this amount to a pint in a very short time. A *catarrhal* intermittent has been admitted by many writers, as also an intermittent *diarrhœa*. A remarkable instance of diabetes has already been noticed by our author, as evidently dependent on a neuralgic affection of the kidneys, which continues to come on periodically even yet, after six years' duration.

"The attacks, during this whole period, were, on every occasion, as strictly periodical as they had ever been, though the relapses became gradually irregular in recurrence and duration both, as happens in all the very chronic cases of these disorders. Nor, in any instance, did the commencement of the saccharine secretion differ from what had been the former hour of the attack of the intermittent, or proceed beyond its ancient limits; occupying six hours of the day. In the interval of the paroxysms, the secretion was perfectly natural, as it was in the greater intervals of the relapses." 143.

These and other examples show, Dr. M. thinks, that neuralgic affections, extending to the nerves which supply the glands, can influence the secretions of those glands, merely in the way of *increase of action*, the case of diabetes forming an exception to the general rule. The facts, however, on which this theory rests, are rather too few, as yet.

"If the neuralgic action on the nerves, and subsequently on the vessels of the few glands that have been noticed, can produce the effects that we have here seen, and if, further, it can produce those very singular inflammations that will hereafter be pointed out, we have a pathological power, the extent and action of which, as exerted on different parts of the compound animal structure, we can imagine to be both various and considerable; a possible cause of diseases the nature of which is still obscure, and a solution of difficulties, which, if it still stops where all pathological investigations do, advances us yet a certain step in this difficult inquiry." 145.

### NEURALGIA FROM INJURIES.

The 8th chapter of the work before us is dedicated to the above subject. But we have not space to dwell upon it, especially as the author confesses that he has few facts of his own to produce, and as we have, in many parts of this Journal, given the results of modern experience on neuralgia from injuries. The first, as well as the most pointed, case occurring in our author's experience, we shall quote in this place.

"The patient was a young woman, and the injury was simply the prick of a needle in the end of the middle finger; its consequence being a regular periodical Neuralgia in that finger; which also did not occur till a few days after the accident. As some weeks had elapsed before she applied for advice, it was easy to ascertain the fact of the periodical and quotidian regularity; while, at that time, the affection of the nerve extended all the way to the shoulder, exactly in the same manner as it does in sciatica and in other cases of spontaneous Neuralgia. And as, further, some time elapsed before I could effect the cure, I had abundant opportunity of satisfying myself respecting the real nature of the disease. That it was cured by means of arsenic, will perhaps, to many physicians, be an additional proof that I had judged correctly respecting its nature. I shall only add, as to this case, what may be an useful hint to practitioners in such instances, that the patient herself had no suspicion of the regular or paroxysmal nature of the pain at the time of her application; and that as it was from my own previous views that I was led to make the inquiry, so it was not without much cross-examination that I was enabled to get the information which I afterwards confirmed by my own observation." 152.

In a second case, a bruise on the arm was followed by a regular neuralgia, of a quotidian character, seated in the middle of the radial nerve, and extending to the shoulder. The cure was effected by arsenic.

The third and last case was neuralgia of the toe, quotidian also, and the result of a blow or bruise. The injury was trifling; but the severity and duration of the pain were great, and continued for more than a month, not in the joint but in the very extremity of the toe. The patient was a medical man, and naturally enough, would not take any medicine for the complaint, though urged to do so by our author. The pain lasted nearly the whole of each day, and vanished every evening. The cure, at last, was spontaneous—an event by no means unusual in many neuralgiæ.

### TOOTH-ACHE.

The ninth chapter, on tooth-ache, occupies 80 pages of letter-press. This may seem preposterous. But we entirely agree with Dr. M. in believing that the man who should discover a rapid remedy for a common *catarrh*, would be a greater benefactor to his race than he who should find out a specific for hydrophobia—the most terrible affliction to which humanity is subject. If there is any one disorder on earth—at least in civilized society—which is universal, it is the tooth-ache. Yet, while its frequency has rendered all classes familiar with the evil, the theory and treatment of the disease have been taken almost entirely out of the hands of physic, to be lodged in the hands of those who can hardly be supposed to be the best philosopers. Dr. M. therefore, does not expect to produce conviction, whatever arguments or evidence he may offer in support of the doctrine, that "tooth-ache, in all its modes, is a neuralgia, and nothing more—a variety of the general disorder under consideration."

"There is, in the first place, a term, a name as old as physic; and I need not again say that a successful and popular term is, in itself, philosophy; cause, theory, every thing; or that it stands at least in lieu of all reasoning and all investigation. It is the Toothach; that is sufficient: after the philosophy is established, the remedies follow of course, and, in this case, it is extraction, the amputation of the imagined seat of the disease: the remedy of the Dracos in legislation for the extirpation of moral diseases.

"As an aid to this reasoning, the very ground-work perhaps of the whole, toothach is pain, pain in a tooth; and thus the entire definition, the whole philosophy and theory, are comprised in this sole feature and term. But when this pain is present, the tooth is frequently unsound, diseased, carious: and thus is the opinion that the pain proceeds from the visible disease, more firmly fixed, while this leading variety becomes the type and parent of the whole. Thus, if the tooth is not really carious, it will be so hereafter, or it ought to be so; or it is diseased in some other manner, (ingenious men find out that there is an abscess concealed) or, as even Cullen has determined, 'it is always dependent on some immediate application of acrid matter to the nerves of the teeth,' or, in short, (for who shall find reasons for those who have none?) it is a diseased tooth in some way, because it gives pain, and therefore it must be extracted: while, at this conclusion does Cullen himself arrive." 173.

And if after all, the disease is not in the tooth, but in the jaw, then it must be in the roots, or in the antrum maxillare—or somewhere else—but still it is TOOTH-ACHE, and the tooth, or all the teeth, must be extracted! The following passage, among a thousand others, exhibits an exquisite specimen of our author's sarcastic style.

"It is not here my business to inquire into the utility, or rather the inutility, of a dentist and his operations: nor to encounter, unnecessarily, the prejudices respecting the wonderful value of his various arts, which are entwined with the most invincible feelings of our vain nature. Yet I may question whether he who is an ingenious workman in ivory, he who is a vendor of secret powders and washes, he whose strength of hand is not checked by any superfluous refinement of feeling, or he who knows no more of the human anatomy than that some teeth have one root and some three, is exactly the fit person to reason about the diseases of the human constitution or to be intrusted with their cure; and, most of all, where, to blind habit and mechanical practice, there is occasionally added a small bias of self-interest." 175.

Dr. M. then enters on the investigation of tooth-ache, forming a division of the disease into varieties, upon a plan of his own. The following extract could not, with benefit, be abridged.

"The heads under which toothach is divided, may, for the present purpose, be conveniently rather than correctly, assumed as the following: but they are very indefinite divisions, as they run into each other, and as individual cases present endless modifications.

"It may be rigidly periodical: while, in this case, it may be either combined, or not, with chronic intermittent fever: and further, in this case also, a tooth, or teeth may be sound, or carious, this condition being here considered incidental, not essential. Or, it may be irregular in its paroxysms, under all the same variations; and in both of these varieties, it passes into the common Neuralgia of the face; or, cases occur, in which either name is equally applicable. This is the periodical toothach so familiar to those who have paid the attention to this subject which it deserves, but which is so very often overlooked, not merely by the physician, but very generally, if somewhat more remarkably, by the patient; as, by the former, its passage into highly marked Neuralgia seems equally to have been neglected, and most so when a carious tooth is present.

"In these forms, it is here considered as spontaneous Neuralgia; while, in the next place, it may occur, either casually, or more regularly, that is, under all the variations of that general disease, and traceable in a marked manner to a carious tooth; the pain being fixed in the nervous branch by which that is supplied, or originating in that and spreading from it. In this form it is here referred to Neuralgia arising from an injury to a nerve.

"Further, as happens in cases of common Neuralgia, the excitement of the minuter vessels may be such as to produce temporary and paroxysmal, or, more permanent inflammation in the membranes, or generally about the face; when it becomes rheumatic toothach or rheumatism of the face, (neuralgic inflammation.) The chief varieties here, are produced in the following manner. The general (rheumatic) inflammation may, as I have just suggested, be paroxysmal and periodical, and even regular, or it may be permanent; while it may attend a carious tooth which is pained, or free of pain, or else a sound tooth in a state of pain; and such pains under any mode of regularity or otherwise. It may also be accompanied by a neuralgic pain independent of the general pain of the inflammation, and not in a tooth; in which case, it is plain, it belongs most unquestionably to Neuralgia, though, in the usual practice, ranked with toothach.

"Or, lastly, all other pain but that of the inflammation itself may be wanting; when it becomes, most strictly, what is called rheumatism of the face: this state also, sometimes occurring, even without the presence of a carious tooth. If this rheumatism of the face with separate neuralgic pain should in correctness have been ranked under common 'Tic,' the last division, also, in a more scientific arrangement, should have been reserved for a distinct section: but its obvious affinities and the received usage render it convenient to include it in the present one; particularly as its limits with respect to the former varieties are indefinite." 179.

The rigidly periodical tooth-ache is by no means a rare form of the disease; but its periodicity must be SOUGHT, otherwise it will seldom force itself on the observation of the routine practitioner.

"Any person who chooses to seek for them, will find cases where the periodical return and the paroxysmal duration of toothach are as regular as those of the most regular Neuralgia or Intermittent fever, and under every variation under which these occur. And this is equally true whether there is a carious tooth or not; while, where that is not present, these cases, by occupying a particular spot or region, and by the quality of the pain, pass gradually into the common and regular Neuralgia of the face (Tic) by gradations so insensible as to have no defined boundary." 181.

Dr. M. has laboured, and with considerable success, to shew that neuralgia of the face appears under endless shapes, both as to place, and the nature or intensity of the pain—and, among other forms, in that of tooth-ache.

"Or, to state this more particularly. If the inferior maxillary nerve can be affected with Neuralgia in its trunk, that affection is, admittedly, not limited to any one point. Let us pursue that point as a mathematical fluent. It proceeds along the nerve till it arrives at the place where the ramification is given off to a tooth; it proceeds even into the tooth, and the *name* is then changed to toothach. But change of name is not change of disease: or if it be so, let the opposing assertion define the point in this fluxion, where the cessation takes place and a new element of equation must be adopted, or where a new disease commences. To determine thus, is to be guided by terms, not by facts or reasoning: this is the very empiricism of which I have so often had occasion to complain; unworthy of science, as it has been the eternal obstacle to the progress of physic." 189.

Dr. M. criticises very sharply the various modes of treatment which have been employed for the cure of tooth-ache, more especially excision and extraction of the tooth. "The abettors of this practice must be content to reflect that they hope to pull the disease out together with the tooth."

"Assuredly, no physician, no one capable of reasoning, would attempt, on principle, to remove the pain produced by, and in, a diseased branch of a nerve, by removing the teeth which are supplied from its minuter ramifications; since it would be as if he were to attempt the cure of sciatica by cutting off the toes." 211.

The establishment of a just theory will, Dr. M. thinks, put an end to the practice of tooth-drawing altogether, as far as it is practised for tooth-ache. Extraction, he maintains, cannot prevent the return of the disease, "otherwise than as, in the case of a carious tooth, they may, like stuffing or filling the cavity, prevent the future operations of the exciting cause, which, in the case of exposed nerves, so often induces a fit of the disorder."

"As I defer the question of carious teeth for the present, the simplest case of this nature is that where there is a pain referred to a single sound tooth; in which, if it does sometimes happen that the pain is thus rigidly limited, it more commonly extends along the cheek or jaw, marking the neuralgic action; while, not unfrequently, the real seat of the pain is there, and the tooth suffers only by extension of it, as in the other cases of Neuralgia formerly described.

"When extraction is resorted to for this kind of toothach, there is sometimes a cure, the cause of which I shall hereafter attempt to explain when speaking of carious teeth; and every cure, of course, unfortunately maintains the practice, while it is seldom enquired how often the operation fails. Very commonly, however, it happens, that immediately after extraction, the patient perceives that the pain continues, or is in the next tooth or teeth; in which case the operator is very often unjustly accused of having extracted the wrong one; when a resolute and confiding patient will sometimes submit to a second or a third loss, though even then the pain will continue. It may perhaps continue still in the neighbouring teeth; though more generally, where this pain has been limited as to the teeth themselves, it disappears from the places of those which have been extracted, continuing in the jaw, or in the leading branch of the nerve. The toothach is then sometimes reputed to have been cured; and the pain of the face is attributed to the operation, or to rheumatism, and is expected to subside; but as it would be endless to detail all these events, I shall not dwell on them." 215.

Dr. M. considers that the exposure of the sensible interior of a tooth, or of the nerve, as it is commonly called, which occurs in a perfectly carious tooth, may be an exciting cause of neuralgia in that nerve. Or the nerve may be diseased in that point; in which cases, extraction may cure the disease, effectually and permanently. Another way in which extraction appears, at times, to relieve tooth-ache, is by exciting pain or temporary disorder—or by the shock which the operation and alarm give to the constitution. Such things occasionally effect a cure in other neuralgiæ, and in intermittents. On the other hand, where there is a tendency to neuralgia in the habit—or the disease is chronic, in the form of tooth-ache, &c. the effect of the extraction may be a removal of the fit of pain, or a cure of the particular paroxysm, or series of paroxysms;—while the disease returns again whenever the unknown constitutional causes, or the fresh application of exciting ones, brings it into action. It is a strong argument in favour of our author's doctrine, that carious teeth do not always, or perhaps generally, produce tooth-ache—even when we know that the nerve is exposed, by its sensibility, to stimulating substances. In short, we see thousands of teeth wasting away through years, with the nerves exposed all the time, and yet without tooth-ache. The rational deduction from this is, that the disease does not essentially consist in such exposure or caries, but that something else is necessary—that the said exposure and caries are only occasional causes. But our limits are so near a close that we must abandon all hope of presenting a full analysis of this, the longest chapter in the work. We shall therefore conclude this article with the summary or chief arguments in the words of the author.

"Let me now however, in concluding, sum up the chief arguments as to toothach, in a condensed and somewhat more logical manner, since they will thus be more impressive than under the diffused illustration which I have been obliged to use in explaining the connections of all the varieties of this disorder. That they involve a series of pure syllogisms, *ought* to render them convincing; that this will do so, is a very different assertion.

"Neuralgia is a pain occupying some point in the nerves of the face, among others; and it may occupy any point in any large branch which supplies the teeth, among other nerves of the face. The pain which it produces is the same pain, whatever be the nerve or part of that nerve affected. The pain of toothach is the same pain, and it is seated in an ultimate extremity of the branch which supplies the teeth, or in more. If that pain is not Neuralgia, then it must follow, that although every other point of that nerve, when pained, is suffering from Neuralgia, let that pain exist any where, from the brain even to the extremity, the very last, ultimate, point, thus suffering, suffers from a different disease. Reductio ad absurdum.

"Neuralgia is regularly intermittent and periodical, or it becomes irregular, under various modes, and from causes, of which many are assignable. The same is true of toothach. Neuralgia is often, or perhaps generally, attended by a peculiar constitutional affection, ascertained to be a chronic intermittent, and when highly marked, there is a febrile paroxysm accompanying the pain. All this is true of toothach. Neuralgia alternates with intermittent fever, by relapses; so does toothach: it alternates with it by paroxysms, and toothach does the same. And in both, the types may be quotidian, tertian, and double tertian, or perhaps more: while, in both, the disease may be similarly doubled; or, being a double tertian, may consist of a paroxysm of the pure intermittent on one day, and of the Neuralgia, or of the toothach, on another.

"Neuralgia is attended by heat, and by excitement of the minute arteries, accompanied by general diffused pain, or irritability, or both; and so is toothach. Neuralgia and toothach are united, or simultaneous: or, the pain and the place of the pain may be such, that neither the physician nor the patient can determine what the disease ought to be called. Neuralgia passes into or produces toothach; and, reversely, toothach passes into or produces Neuralgia. The two pains alternate, in various modes; or that which was Neuralgia at one period, be that of day, hour, or minute, or even instant, may be toothach in the next; or the reverse.

"Neuralgia is produced by the injury of a nerve. So is toothach: and this is the case of a carious tooth. And if toothach from this cause is especially frequent, it is that the caries of a tooth is very common; and that there are not, in external circumstances, or in the body, any frequent means of thus injuring, either through accident or disease, the branch of a nerve elsewhere.

"Toothach, when regular, is cured by the same remedies as regular Neuralgia. In many instances it is thus cured even when irregular: and if such cure is not more frequent, it is because physicians have not thus attempted it: not having taken that view of this disease which I have here endeavoured to establish." 244.

We have now got through the first half of the last volume of Dr. M.'s work, and shall be able to conclude the whole, in one more article. It may seem remarkable, that not one of our contemporaries has attempted an *analysis* of the work before us. We ourselves are at no loss for a solution of this enigma. In the course of our lives we have hardly ever encountered a more difficult task than an *analytical* review of Dr. Macculloch's volumes. The reason will be obvious to those, and those only, who peruse the work. We do not regret our labours. The views which the ingenious author has opened out, will shed lustre on his own name, and on the profession to which he belongs, (however severely he may have satirized its professors) long after the spirit has deserted its tenement of clay, and the tenement itself has ceased to retain any mark of its original form or identity.

